# Complete depletion of primordial germ cells in an All-female fish leads to Sex-biased gene expression alteration and sterile All-male occurrence

**DOI:** 10.1186/s12864-015-2130-z

**Published:** 2015-11-18

**Authors:** Wei Liu, Shi-Zhu Li, Zhi Li, Yang Wang, Xi-Yin Li, Jian-Xiang Zhong, Xiao-Juan Zhang, Jun Zhang, Li Zhou, Jian-Fang Gui

**Affiliations:** State Key Laboratory of Freshwater Ecology and Biotechnology, Institute of Hydrobiology, Chinese Academy of Sciences, Graduate University of the Chinese Academy of Sciences, Wuhan, 430072 China

**Keywords:** Sexual dimorphism, Gonad differentiation, Primordial germ cells, Gynogenesis, Sex-biased gene, *Carassius gibelio*

## Abstract

**Background:**

Gynogenesis is one of unisexual reproduction modes in vertebrates, and produces all-female individuals with identical genetic background. In sexual reproduction vertebrates, the roles of primordial germ cells on sexual dimorphism and gonadal differentiation have been largely studied, and two distinct functional models have been proposed. However, the role of primordial germ cells remains unknown in unisexual animals, and it is also unclear whether the functional models in sexual reproduction animals are common in unisexual animals.

**Results:**

To solve these puzzles, we attempt to utilize the gynogenetic superiority of polyploid *Carassius gibelio* to create a complete germ cell-depleted gonad model by a similar morpholino-mediated knockdown approach used in other examined sexual reproduction fishes. Through the germ cell-depleted gonad model, we have performed comprehensive and comparative transcriptome analysis, and revealed a complete alteration of sex-biased gene expression. Moreover, the expression alteration leads to up-regulation of testis-biased genes and down-regulation of ovary-biased genes, and results in the occurrence of sterile all-males with testis-like gonads and secondary sex characteristics in the germ cell-depleted gynogenetic *Carassius gibelio*.

**Conclusions:**

Our current results have demonstrated that unisexual gynogenetic embryos remain keeping male sex determination information in the genome, and the complete depletion of primordial germ cells in the all-female fish leads to sex-biased gene expression alteration and sterile all-male occurrence.

**Electronic supplementary material:**

The online version of this article (doi:10.1186/s12864-015-2130-z) contains supplementary material, which is available to authorized users.

## Background

Primordial germ cells (PGCs), the ancestors of sperms and eggs, arise before the formation of gonadal somatic cells and migrate to genital ridge, in which the PGCs and somatic cells form primordial gonad [1, 2]. Then, the primordial gonad differentiates into a testis or an ovary under their collaboration [3]. In mammals, the sexual fate is determined by a Y-linked *Sry* gene, which initiates a cascade of events to trigger the primordial gonads to differentiate into testes [4]. And, the *Sry* expression in common precursors also triggers differentiation of the somatic precursors into Sertoli cells [5]. In Japanese medaka, a Y-specific *Dmy*, which is expressed in the gonadal somatic cells of XY embryos, has been revealed to make the gonads differentiate into testes [6]. Moreover, several sex determination-related or sex chromosome-linked genes have been identified, and most of them are the duplications of *dmrt1* (dsx and mab-3 related transcription factor 1) [7–10]. As primordial gonad is composed of PGCs and somatic precursors, and gonadal differentiation and gametogenesis must go through a long and complicated developmental process, the interaction between germ cells and somatic cells is therefore very critical for the process completion [11].

In mammals, the germ cell-depleted XY mouse embryos were not found to affect the ability of supporting cells to develop into testicular cords [12], whereas in XX mouse, germ cell ablation before birth did not affect the ovary development [13]. Moreover, through losing sex determination-related gene *dmrt1* in mature testis or by depleting female determination-related gene *foxl2* in mature ovary, the gonadal somatic cell sex was also demonstrated to be required for testis or ovary maintenance throughout adulthood [14, 15]. More complicated roles of germ cells on gonad differentiation and sexual dimorphism had been observed in teleost fish and reptilian turtle. In Japanese medaka, Kurokawa et al. [16] revealed that loss of germ cells in XX medaka resulted in a failure to maintain female supporting cells and the somatic cells acquired male supporting cell characteristics, in which the produced androgens made the germ cell-depleted medaka undergo a female-to-male sex reversal in secondary sex characteristics. In zebrafish, the germ cell-depleted fish were demonstrated to be males, and the oocytes were confirmed to be required for a stable maintenance of sexual phenotype in adults [17–19]. Moreover, the number of germ cells was also demonstrated to contribute to sex differentiation and gonad dimorphism in zebrafish and medaka, in which the embryos with a number of germ cells lower than a threshold develop into males, while those with plenty of germ cells become females [20–22]. These results in zebrafish and medaka seem to indicate that germ cells play an active role in regulating gonad differentiation and sexual dimorphism. However, in other fish species such as loach and goldfish, loss of germ cells was not revealed to alter dimorphic gonadal structure and even gene expression [23, 24], and in red-eared slider turtle, the loss of germ cells was not observed to affect the morphogenesis of fetal ovary or testis [25], implicating that germ cells might be not primary for sex differentiation and sexual dimorphism. The above data indicate that there are two distinct functional models of germ cells on sexual dimorphism and gonadal differentiation in sexual reproduction vertebrates. In vertebrates including fish, amphibians and reptiles, about 90 species have been reported to contain all-female unisexual forms, and these unisexual vertebrates have been demonstrated to reproduce by gynogenesis, hybridogenesis, parthenogenesis, or kleptogenesis [26–31]. As one of unisexual reproduction modes, gynogenesis is able to produce all-female individuals with the same genetic background, because the all-females are generated only from the maternal nucleus. However, whether the developing embryos originated maternal nucleus by gynogenesis are able to develop into males or not remain completely unknown, and the roles of germ cells on sex determination and gonad differentiation are quite unclear in the unisexual animals. Therefore, more studies need to be further performed in some unisexual reproduction models.

*Carassius gibelio*, a polyploid cyprinid fish, has been revealed to have multiple reproduction modes including sexual reproduction and unisexual gynogenesis [32–35]. When its mature eggs are stimulated by heterologous sperm from the male of another species, such as *Cyprinus carpio*, the activated eggs develop into all-female polyploid offspring by a typical unisexual gynogenesis. Previous studies have indicated that all embryos and all individuals resulted from gynogenesis in polyploid *Carassius gibelio* possess the same genetic background, as they are generated only from the maternal female nucleus [30, 36, 37]. To further investigate the role of germ cells on gonad differentiation and sexual dimorphism fate, here, we attempted to utilize the gynogenetic superiority of polyploid *Carassius gibelio* to create a complete germ cell-depleted gonad model by a similar approach used in other examined sexual reproduction fishes [16, 17, 23, 24]. Firstly, the complete germ cell-depleted gonad model was established by morpholino-mediated knockdown of *dnd* (*dead end*), an essential factor for PGC migration and survival [38]. Using this model, we observed the gonadal tissue structure changes throughout gonad differentiation. And, the germ cell-depleted gonads at different development and growth stages were subjected to comparative transcriptome analysis to pursue expression alteration of gonadal sex-biased genes. Moreover, the altered consequences including secondary sex characteristics and gonadal structure changes were further investigated in the complete germ cell-depleted adults from 1 year to 3 years. These comprehensive investigations have not only confirmed the leading effect of germ cells on gonadal differentiation and sexual dimorphism, but also found that the complete depletion of primordial germ cells in the all-female polyploid fish leads to sex-biased gene expression alteration and sterile all-male occurrence.

## Results

### Establishment of complete germ cell-depleted gonad model in gynogenetic *Carassius gibelio*

To create complete germ cell-depleted gonad model, we firstly cloned a germ cell marker gene *dnd* from *Carassius gibelio*. The *dnd* (accession number KP641680) is highly conserved, and the predicted amino acid sequence shares 34 to 92.8 % identities with other vertebrate orthologues (Additional file 1: Figure S1). Using an antisense morpholino (MO) strategy, a *dnd*-specific morpholino oligonucleotide (*dnd*-MO: 5’- AGCTGCTGTCCCTCCATACCGCTGT-3’) that specifically targets translation start codon of *Carassius gibelio dnd* transcript was designed and injected into early gynogenetic one-cell stage embryos activated by heterologous sperm of red common carp. The efficiency of PGC depletion was examined by detecting *vasa* mRNA, an important factor for tracing PGC migration in vertebrates [39–41]. The data indicate that *dnd* is essential for PGC survival and proliferation in the gynogenetic embryos, because no any PGCs are observed in the 24 hpf *dnd*-MO embryos (Additional file 2: Figure S2). Moreover, we analyzed the sensitive dosage of the *dnd*-MO injection, and revealed that the complete depletion of PGCs was generated as the injected *dnd*-MO dosage was up to 1000 pg/embryo, in which no any germ cells were observed at the 24 hpf embryos (Additional file 2: Figure S2).

**Fig. 1:**
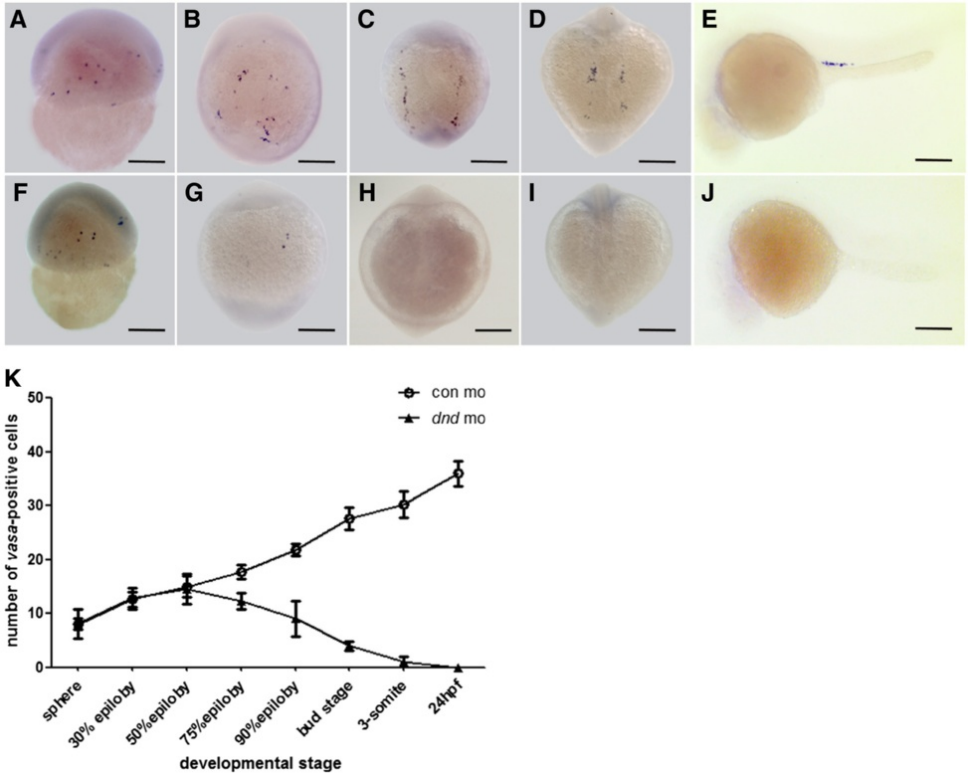
Identification and distribution of *vasa*-positive PGCs by whole mount *in situ* hybridization in WT (**a**-**e**) and *dnd*-MO injected (2000 pg/per embryo) (**f**-**j**) embryos from 50 % epiboly to 36 hpf. **a** and **f** 50 % epiboly stage, **b** and **g** bud stage, **c** and **h** 3-somite stage, **d** and **i** 24 hpf, and **e** and **j** 36 hpf. (Scale bar, 250 μm). **k** Average number of *vasa*-positive PGCs/per embryo in embryos injected with con-MO (circle) or *dnd*-MO (triangle) from sphere to 24 hpf. N ≥ 30, and the statistical data are presented as mean ± standard deviation (SD), *P* < 0.001

Then, we used 2000 pg/embryo dosage to create complete germ cell-depleted gonad model and comparatively traced the existence and migration status of PGCs in wild type (WT) and the *dnd*-MO injected gynogenetic embryos. As shown in Fig. 1, at 50 % epiboly, PGCs are gathered into three clusters at the marginal region of both WT and the *dnd*-MO injected gynogenetic embryos (Fig. 1a and f). At bud stage, PGCs are widely distributed on the dorsal side in wild type gynogenetic embryos, whereas their number is dramatically reduced in the *dnd*-MO injected gynogenetic embryos (Fig. 1b and g). At 3-somite stage, numerous PGCs are aggregated into two lines along the junction of yolk extension in WT gynogenetic embryos, while almost no any PGCs are observed in the *dnd*-MO gynogenetic embryos (Fig. 1c and h). In WT gynogenetic embryos at 24 hpf and 36 hpf, the dispersed PGCs on both sides of the axis are migrated and concentrated onto the 7-9th somite along the anterior area of yolk extension (Fig. 1d and e), however, no any PGCs are found in 99.25 % of the corresponding *dnd*-MO injected gynogenetic embryos (Fig. 1i, j; Additional file 3: Table S1). Moreover, we analyzed the number of PGCs in each embryo of the *dnd*-MO and WT embryos. As shown in Fig. 1k, before 50 % epiboly, the *dnd*-MO embryos have an equal number of PGCs to that in WT embryos, whereas the average number is rapidly reduced from 70 % epiboly, and almost no any PGCs are observed at 3-somite stage from the *dnd*-MO embryos. These data indicate that the *dnd* translation block results in complete depletion of PGCs, and significantly, the completely germ cell-lost embryos and fingerlings are still able to survive to adulthood, in which a germ cell-depleted gonad model is thereby established in the gynogenetic fish.

### Gonadal and histological structure changes during gonad differentiation in the germ cell-depleted gynogenetic *Carassius gibelio*

**Fig. 2:**
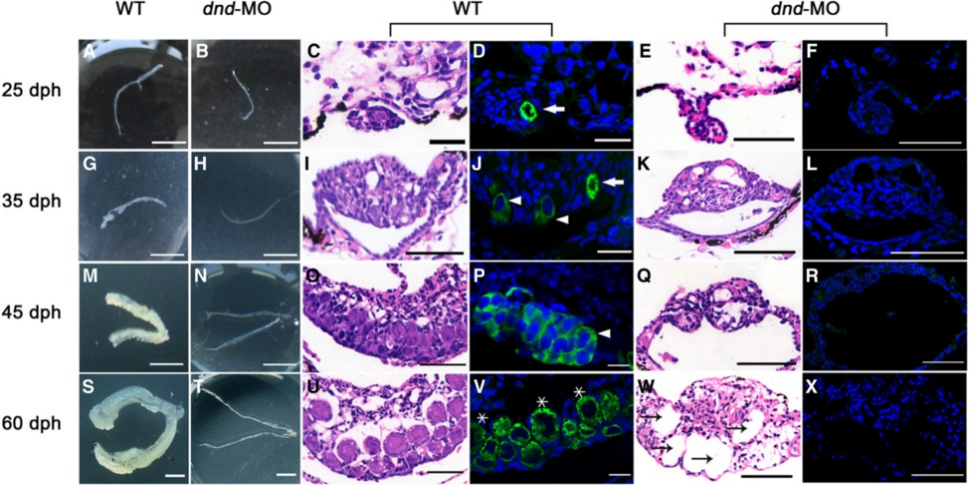
Gonadal and histological structures of WT and *dnd*-MO gynogenetic fish during gonadal differentiation. Gonadal and histological structures of WT and *dnd*-MO gonads at 25 dph (**a**-**f**), 35 dph (**g**-**l**), 45 dph (**m**-**r**) and 60 dph (**s**-**x**). The external morphology of WT gonads (**a, g, m** and **s**), and *dnd*-MO gonads (**b, h, n** and **t**). Haematoxylin–eosin and immunofluorescence staining of WT gonads (**c, d, i, j, o, p, u** and **v**) and *dnd*-MO gonads (**e, f, k, l, q, r, w** and **x**). White arrow indicates PGC, arrow head indicates differentiating oogonia, star indicates primary oocyte and black arrow indicates cavity. [Scale bars, 500 mm (**a, b, g** and **h**), 1 cm (**m, n, s** and **t**), 20 μm (**c** and **d**) and others 50 μm

Gonad differentiation and PGC proliferation of *Carassius gibelio* were previously observed to occur from 10 to 30 day post-hatching (dph) juveniles [42]. To trace gonadal and histological structure changes during gonad differentiation, the *dnd*-MO and the corresponding WT gynogenetic juveniles were anatomized at 25, 35, 45, and 60 dph, and their cytological observation was performed by HE (haematoxylin–eosin) staining and Vasa immunofluorescence localization as described [43]. In comparison with WT gonads with PGCs at 25 dph (Fig. 2a, c and d), the *dnd*-MO gonads were observed to be thin (Fig. 2b), and no any PGCs were found in the histological sections (Fig. 2e and f). At 35 dph, the differentiating oogonia were seen in WT gonads with PGCs (Fig. 2g, i and j), whereas no germ cells including PGCs were observed in the *dnd*-MO gonads (Fig. 2h, k and l). As numerous oogonia were differentiated and proliferated in the enlarged WT gonads at 45 dph (Fig. 2m, o and p), there were still no any PGCs and germ cells in the corresponding *dnd*-MO gonads without any external changes, and only some empty cavities were formed in the gonadal tissues (Fig. 2n, q and r). At 60 dph, the WT gonads had developed into typical ovaries (Fig. 2s) with a lot of primary oocytes (Fig. 2u and v). However, the corresponding *dnd*-MO gonads remained in the thin thread-like structures (Fig. 2t), where only gonadal somatic cells were surrounded around the enlarged cavities (Fig. 2w and x). These data indicate that gonad and germ cell differentiation has lasted in WT gynogenetic *Carassius gibelio* juveniles from 25 dph to 60 dph, and significant gonadal and histological structure changes have occurred in the corresponding *dnd*-MO gonads, in which WT gonads choose female sexual fate and differentiate into ovaries with numerous oogonia and primary oocytes, whereas the germ cell-depleted gonads undergo severe differentiation and development damage.

### Complete depletion of germ cells alters sex-biased gene expression during gonad differentiation

**Fig. 3:**
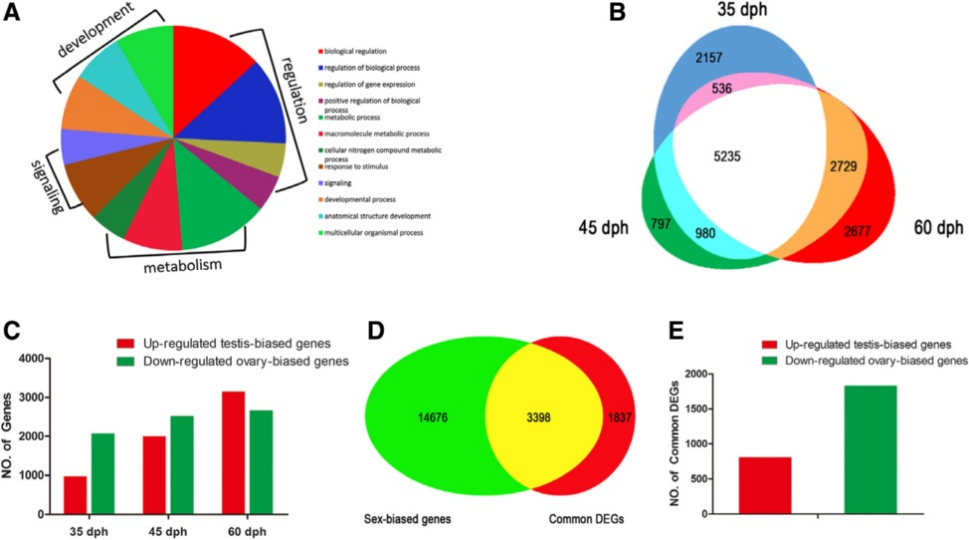
Expression changes of sex-biased genes during gonad differentiation. **a** Pie charts representing the main GO terms (biological process) associated with the DEGs in germ cell-depleted gonads. Only GO terms with >800 DEG sequences are shown (Except for DEGs in germ cell-depleted gonads at 25 dph). **b** Venn diagram shows the commonly and differentially DEGs existed in the germ cell-depleted gonads at 35 dph, 45 dpg and 60 dph. **c** Sex-biased gene expression patterns in germ cell-depleted gonads in comparison to WT gonads from 35 dph to 60 dph. **d** Venn diagram shows the overlap association between common DEGs in germ cell-depleted gonads from 35 dph to 60 dph and sex-biased genes in normal testis and ovary. **e** Expression pattern of sex-biased genes in common DEGs in germ cell-depleted gonads from 35 dph to 60 dph

Subsequently, we performed comparative transcriptome analysis to investigate the expression characteristics and differentially expressed genes (DEGs) in germ cell-depleted gonads at above corresponding differentiation stages. In comparison with WT gonads during gonad differentiation, a total number of 942, 8908, 9741 and 11177 DEGs were respectively revealed in germ cell-depleted gonads at 25 dph, 35 dph, 45 dph and 60 dph (Additional file 4: Figure S3A-D; Additional file 5: Table S2). And, gene ontology (GO) classification analysis revealed similar distribution patterns of the above DEGs among 45 GO terms at 25, 35, 45 and 60 dph in germ cell-depleted gonads in comparison with WT gonads (Additional file 6: Figure S4A-D). Moreover, some associated DEGs in the germ cell-depleted gonads were used to perform Pie chart analysis, and they were further classified into four main biological processes, such as regulation, metabolism, signaling and development (Fig. 3a). To identify whether the DEGs between germ cell-depleted gonads and WT gonads during gonad differentiation were sex-biased genes, we further performed comprehensive transcriptome analysis of mature ovary and testis (the male resulted from sexual reproduction of *Carassius gibelio* [30, 37]). A total of 18074 sex-biased genes were obtained (Additional file 7: Table S3), in which 13765 were testis-biased genes and 4309 were ovary-biased genes (Additional file 4: Figure S3E, F).

As gonads at 25 dph were undifferentiated (Fig. 2a-d), and thus, we mainly compared the overall DEGs in the germ cell-depleted gonads relative to WT gonads from 35 dph to 60 dph. As shown in Fig. 3b, in comparison with corresponding WT gonads, there exist 5235 common DEGs in the germ cell-depleted gonads from 35 dph to 60 dph, and there are 2157, 797 and 2677 DEGs to be only found in germ cell-depleted gonads at 35 dph, 45 dph, 60 dph, respectively. Additionally, there are 980 DEGs to coexist in germ cell-depleted gonads at 35 dph and 45 dph, 2729 DEGs to coexist in germ cell-depleted gonads at 45 dph and 60 dph, and 536 DEGs to coexist in germ cell-depleted gonads at 35 dph and 60 dph. And, a comparative analysis was carried out between the DEGs in the germ cell-depleted gonads and sex-biased genes. Along with gonad differentiation, a total number of 975, 1998 and 3145 testis-biased genes are respectively up-regulated in germ cell-depleted gonads at 35 dph, 45dph and 60dph, whereas 2066, 2522 and 2661 ovary-biased genes are down-regulated in germ cell-depleted gonads at 35 dph, 45dph and 60dph, respectively (Fig. 3c). Furthermore, we examined the overlap association between 5235 common DEGs of germ cell-depleted gonads from 35 dph to 60 dph and sex-biased genes in normal testis and ovary. As a result, about 65 % (3398) common DEGs were sex-biased genes (Fig. 3d), in which 807 testis-biased genes were continuously up-regulated in germ cell-depleted gonads in comparison to WT gonads from 35 dph to 60 dph, whereas 1830 ovary-biased genes were down-regulated in germ cell-depleted gonads in comparison to WT gonads (Fig. 3e).

Additionally, some sex differentiation-related genes [44, 45] were searched through our Blast and GO data (Additional file 5: Table S2; Additional file 7: Table S3). Thereby, a total of 24 sex differentiation-related genes were revealed within the assembled transcriptomes. As shown in Table 1, there are 18 up-regulated genes in germ cell-depleted gonads, in which most (16) of them are testis differentiation-related genes, and other two are *gata4* and *wt1a* genes expressed in bipotential gonad, whereas all 6 down-regulated genes in germ cell-depleted gonads are ovary differentiation-related genes.

**Table 1:** Up-regulated or down-regulated expression of 24 sex differentiation-related genes in the germ cell-depleted gonads

Hit sequence ID	Species	Gene name	Gene description	Gene	Up or down-regulation	
					Exp.	Obs.
EU136185.1	squalius pyrenaicus	*amh*	anti-Mullerian hormone	Unigene7325	T	+++
AY090897.1	goldfish	*ar*	androgen receptor	Cl14013.c2	T	+++
NM_001080204.1	black porgy	*cyp11b*	11-beta-hydroxylase	Unigene57084	T	+++
ACT79291.1	squalius alburnoides	*dax1*	nuclear receptor subfamily 0 group B member 1	Unigene6035	T	+++
JQ413415.1	zebrafish	*dmrt1*	doublesex- and mab-3 related transcription factor 1	Cl355.c2	T	+++
NM_001005779.2	zebrafish	*dmrt3*	doublesex and mab-3 related transcription factor 3	Unigene44639	T	+++
NM_001114668.1	zebrafish	*gsdf*	gonadal soma derived factor	Cl9466.c1	T	+++
AAF43283.1	zebrafish	*nr5a1a*	nuclear receptor subfamily 5, group A, member 1a	Cl40.c1	T	+++
BC078289.1	zebrafish	*pdgfaa*	platelet derived growth factor alpha a	Cl1048.c1	T	+++
NM_001076757.1	zebrafish	*pdgfab*	platelet derived growth factor alpha b	Cl14024.c1	T	+++
Z32814.1	zebrafish	*pdgfra*	platelet-derived growth factor receptor, alpha	Cl11648.c2	T	+++
NP_001070225.1	zebrafish	*pdgfrb*	platelet-derived growth factor receptor beta	Unigene9549	T	+++
XM_860152.2	loach	*sox8*	SRY-related HMG-box 8	Unigene61836	T	+++
AAG09814.1	zebrafish	*sox9a*	SRY-related HMG-box 9	Cl11561.c1	T	+++
AY956415.1	goldfish	*sox9b*	SRY-related HMG-box 9	Unigene11888	T	+++
JQ217143.1	loach	*sox10*	SRY-related HMG-box 10	Cl19883.c1	T	+++
DQ886664.1	zebrafish	*gata4*	GATA-binding protein 4	Cl12384.c1	B	+++
BC162638.1	zebrafish	*wt1a*	wilms tumor suppressor 1a	Cl4953.c1	B	+++
BC056276.1	zebrafish	*ctnnb1*	catenin β-1	Unigene4098	O	---
AF020704.1	goldfish	*cyp19a1a*	aromatase a	Unigene14479	O	-
AB531497.1	loach	*foxl2*	forkhead box transcription factor L2	Cl17424.c1	O	-
NM_001039621.1	zebrafish	*fst*	Follistatin	Unigene1876	O	---
AAI24452.1	zebrafish	*srd5a1*	3-oxo-5-alpha-steroid 4-dehydrogenase 1	Cl17085.c2	O	---
NM_001044939.1	zebrafish	*srd5a3*	3-oxo-5-alpha-steroid 4-dehydrogenase 3	Cl18471.c2	O	---

Exp.: The expected over-expression in testis (T), bipotential gonad (B) or ovary (O) of other vertebrate species; Obs.: The observed up-regulated or down-regulated expression in germ cell-depleted gonads compared with WT gonads. “+++”: Significantly up-regulated expression; “---”: Significantly down-regulated expression; “-”: mildly down-regulated expression

The above comprehensive and comparative transcriptome data indicate that complete depletion of germ cells alters sex-biased gene expression and thereby leads to up-regulation of testis-biased genes and down-regulation of ovary-biased genes in the germ cell-depleted gonads of gynogenetic *Carassius gibelio*.

### Testicular differentiation-related genes are significantly up-regulated in the germ cell-depleted gonads

**Fig. 4:**
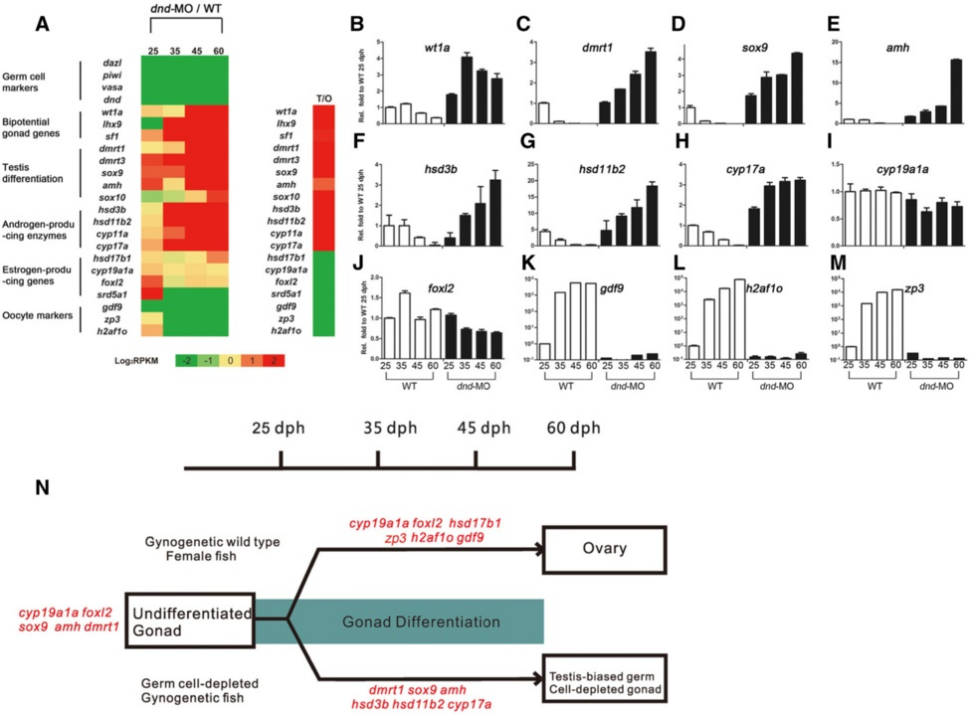
Testis differentiation-related genes are up-regulated in *dnd*-MO gonads during gonadal differentiation. **a** Transcriptomic analysis of gonadal development-related genes expressing in WT and *dnd*-MO gonads from 25 dph to 60 dph (left) and in mature testis relative to mature ovary (right). **b**-**i** RT-PCR detection of the indicated genes above. **b** *Wt1a*, **c** *dmrt1*, **d** *sox9*, **e** *amh*, **f** *hsd3b*, **g** *hsd11b2*, **h** *cyp17a*, **i** *cyp19a1a*, **j** *foxl2*, **k** *gdf9*, **l** *h2af1o* and **m** *zp3*. **n** Schematic diagram of gene expression pathways during gonadal differentiation from 25 dph to 60 dph in WT gynogenetic fish and the germ cell-depleted gynogenetic fish. The gonadal differentiation-related genes (red) are shown in the corresponding positions

Moreover, we analyzed and compared dynamic expression patterns of several kinds of sex-related marker genes involved in gonad development between WT and the corresponding germ cell-depleted gonads during gonad differentiation. As expected, four germ cell marker genes, such as *dazl*, *piwi*, *vasa*, and *dnd* [3, 40, 42, 46], were revealed to be absent in the germ cell-depleted gonads (Fig. 4a, left). Dynamic comparative transcriptome analysis showed that bipotential gonad development genes: *wt1a* and *sf1* [47], testis-differentiation genes, such as *dmrt1*, *dmrt3*, *sox9*, *amh* and *sox10* [41, 43, 45, 48, 49], and some steroidgenic enzyme genes involved in active androgen biosynthesis such as *hsd3b*, *hsd11b2*, *cyp11a* and *cyp17a* [50–54], were significantly up-regulated in germ cell-depleted gonads during gonad differentiation (Fig. 4a, left). In contrast with the above dynamic expression changes of testicular differentiation and steroidgenic enzyme-related genes, some genes required for estrogen reproduction and ovary differentiation, such as *hsd17b1*, *cyp19a1a* and *foxl2* [53–55], were mildly down-regulated, and some oocyte markers including *gdf9*, *zp3* and *h2af1o* [56–60] were dramatically down-regulated in the germ cell-depleted gonads in comparison to WT gonads (Fig. 4a, left). Consistent with the gene expression changes, these sex-related genes had similar expression patterns in mature testis in comparison with mature ovary (Fig. 4a, right).

Furthermore, the expression changes of these sex-related genes were examined by RT-PCR detection. *Wt1a*, expressed in the bipotential gonad, was expressed in WT gonads and up-regulated in germ cell-depleted gonad (Fig. 4b). *Dmrt1*, *sox9* and *amh* were observed to express in both early WT and the germ cell-depleted gonads at 25 dph, but obvious down-regulated expression in WT gonads and significant up-regulated expression in the corresponding germ cell-depleted gonads were found at later stages of 45 dph and 60 dph (Fig. 4c-e). In addition, *hsd3b*, *hsd11b2* and *cyp17a*, were also revealed to have dynamic expression changes similar to that of testicular differentiation-related marker genes, in which obvious down-regulated expression in WT gonads and significant up-regulated expression in the corresponding germ cell-depleted gonads were observed along with the gonad differentiation progress (Fig. 4f-h). In contrast, *cyp19a1a* and *foxl2*, the ovary differentiation genes, were expressed in both WT and germ cell-depleted gonads at 25 dph, and up-regulated at 35 dph in WT gonads and maintained high expression levels in later stages, but obviously down-reguleted in germ cell-depleted gonads (Fig. 4i and j). Significantly, the oocyte marker genes including *gdf9*, *h2af1o* and *zp3* were found to increase dramatically in WT gonads as the gonads were differentiated into ovaries, whereas only a little of their expression products were detected in the later germ cell-depleted gonads (Fig. 4k-m).

On the bases of transcriptomic analysis and RT-PCR detection, we summarized the gene expression pathways of gonadal development in WT gynogenetic fish and germ cell-depleted fish. As shown in Fig. 4n, in gynogenetic *Carassius gibelio*, there exist a certain of transcripts of testis and ovary differentiation-related genes, such as *dmrt1*, *sox9*, *amh*, *cyp19a1a* and *foxl2*, in both WT and germ cell-depleted undifferentiated gonads. As gonadal development, the testis differentiation-related genes including *dmrt1*, *sox9* and *amh* are rapidly down-regulated, and the ovary differentiation-related genes including *cyp19a1a*, *foxl2*, *hsd17b1* and oocyte marker genes including *zp3*, *h2af1o* and *gdf9* are up-regulated, and thereby trigger follicle development to lead the WT gonad to ovary. In contrast to WT gonad, the ovary differentiation-related genes are down-regulated, whereas the testis differentiation-related and androgen producing genes, such as *dmrt1*, *sox9*, *amh*, *hsd3b*, *hsd11b2* and *cyp17a*, are up-regulated, and make the germ cell-depleted gonad differentiate into testis-like tissue.

### Phenotypic masculinization occurs in the germ cell-depleted gynogenetic adults

**Fig. 5:**
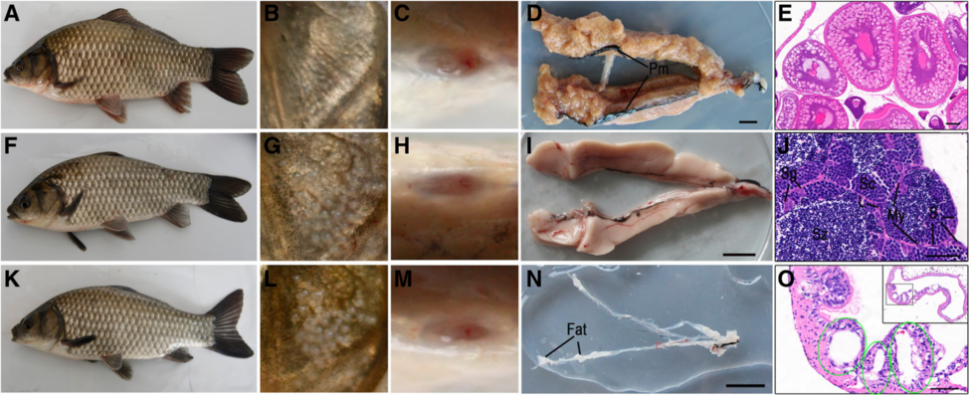
Phenotypic masculinization and their gonadal morphology in the germ cell-depleted adults. **a**-**e**, **f**-**j** and **k**-**o** Representative images of control gynogenetic females, control males from sexual reproduction, and the germ cell-depleted gynogenetic males, respectively. **a, f** and **k** Body shape. **b, g** and **l** Gill cover. **c, h** and **m** Anus. **d, i** and **n** Images of mature ovary, testis, and 1-year-old germ cell-depleted gonad, respectively. **e, j, o** HE staining of control ovary, control testis, and 1-year-old germ cell-depleted gonad, respectively. Green circle indicates the cavity, inset shows the whole cross section of 1-year-old germ cell-depleted gonad. Pm, Peritoneal membrane; Sg, spermatogonia; Sc, spermatocytes; Sz, spermatozoa; L, Leydig cells; S, Sertoli cells; My, peritubular myoid cells. [Scale bars, D, I and N, 2 cm; E 100, μm, J and O 50 μm]

To comparatively investigate masculinization occurrence in the germ cell-depleted adults, we simultaneously used sexual reproduction mode [32, 37] to produce normal male and female individuals in this study, because adult males and females can be distinguished by their external characteristics including body shape, pearl organs and anus. As shown in Fig. 5, during breeding season, the 1-year adult females are fat and have round anus but no pearl organs, and develop into mature ovaries that contain different stage oocytes (Fig. 5a-e), whereas the 1-year adult males are slender with lots of pearl organs on the gill cover and have prolate anus, and form mature testis with spermatogenic cysts and numerous sperms (Fig. 5f-j). In comparison with these normal control female and male adults, the 1-year germ cell-depleted adults show normal male secondary sex characteristics, in which all of them present slender body shape and have pearl organs and prolate anus (Fig. 5k-m; Additional file 8: Table S4). In contrast with full-grown mature ovaries (Fig. 5d, e) and testes (Fig. 5i, j) in normal females and males, almost all of the germ cell-depleted adults develop transparent tube-like structures (Fig. 5n), in which only some cavities are surrounded by gonadal somatic cells (Fig. 5o). These observations indicate that phenotypic masculinization and female-to-male sex reversal have occurred in the germ cell-depleted gynogenetic adults.

### The germ cell-depleted gynogenetic adults develop testis-like gonads without spermatogenic cells

**Fig. 6:**
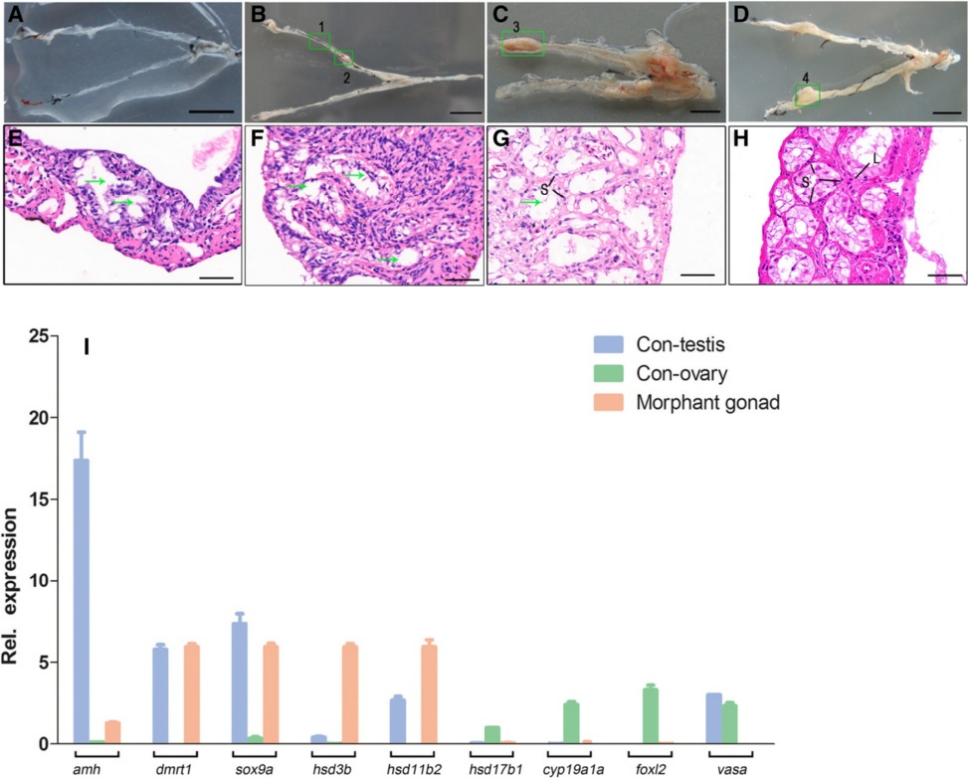
Gonadal structures and gene expression patterns in adult germ cell-depleted gynogenetic gonads. **a**-**d** The external morphology of 1 year to 3 years old germ cell-depleted gonads. **a** 1-year-old germ cell-depleted gonads. **b** 2-year-old germ cell-depleted gonads. **c** and **d** 3-year-old germ cell-depleted gonads. **e**-**h** Cross section of germ cell-depleted gonads in above box 1 (**e**), 2 (**f**), 3 (**g**) and 4 (**h**). Green arrow indicated the cavity. L, Leydig cells; S, Sertoli cells. [Scale bars, 2 cm (**a-d**) and 50 μm (**e**-**h**). **i** RT-PCR analysis of somatic gene expression in control testis (Con-testis) from sexual reproduction, control ovary (Con-ovary) and the 3-year-old germ cell-depleted gonad (Morphant gonad). The gene expression level is related to β-actin and *Rpl13a*

Furthermore, these germ cell-depleted gynogenetic adults were allowed to grow for three years, and their gonads were anatomized and reexamined from 1 to 3 year-old adults. As shown in Fig. 6, in the 1-year-old and 2-year-old germ cell-depleted gynogenetic adults, most of them (17/18 in 1-year-old and 15/18 in 2-year-old adults) have transparent tube-like gonadal structures (Fig. 6a), whereas only a few of individuals (1/18 in 1-year-old and 3/18 in 2-year-old adults) show partly enlarged tube-like gonadal structures (Fig. 6b). Along with further growth, about half of the tube-like gonads (9/17) become thick and partly enlarged in the 3-year-old germ cell-depleted gynogenetic adults (Fig. 6c, d).

Histological observations show that both the transparent tube-like and partly enlarged portions in the 2-year-old germ cell-depleted gonads include numerous cavities with more somatic cells in the enlarged portions (Fig. 6b, e and f). In the thick and partly enlarged gonads of the 3-year-old germ cell-depleted gynogenetic adults (Fig. 6c, d), the gonadal somatic cells represent regular arrangement, in which they develop into more empty cavities surrounded by Sertoli cells (Fig. 6g), and sometimes, these cavities and the surrounded gonadal somatic cells constitute spermatogenic cyst-like structures in which numerous Sertoli cells and Leydig cells can be distinguished, but there are no any spermatogenic cells (Fig. 6h). Therefore, in the adult germ cell-depleted gonads, gonadal somatic cells differentiate into Sertoli cells and Leydig cells, which are reorganized into testicular structures.

Moreover, we reexamined several marker gene expression patterns of the 3-year-old germ cell-depleted gonads relative to the corresponding normal ovaries and testes by RT-PCR analysis. As shown in Fig. 6i, three Sertoli cell markers genes including *amh*, *dmrt1* and *sox9a* are abundantly expressed in the 3-year-old germ cell-depleted gonads and the control testes, although the *amh* expression level is lower in the 3-year-old germ cell-depleted gonads than in the control normal testes. Even the steroidgenic androgen enzyme genes *hsd3b* and *hsd11b2* are more highly expressed in the 3-year-old germ cell-depleted gonads than in the control normal testes, and there is no any expression in the control normal ovaries, whereas the estrogen-producing enzyme genes *hsd17b1* and *cyp19a1a* and ovary marker gene *foxl*2 are expressed in the control normal ovaries but not in the germ cell-depleted gonads or control testes, and the germ cell marker gene *vasa* is equally expressed in both of control testes and ovaries, but not in germ cell-depleted gonads (Fig. 6i). Therefore, the germ cell-depleted gonads in the morphant adults not only have testis-like gonad structures but also represent gene expression pattern of testicular somatic cells similar to WT testes.

## Discussion

Up to the present, two completely different arguments on functional role of germ cells on gonadal development have been suggested in sexual reproduction animals. The first one was proposed from the investigations in mouse, medaka and zebrafish that germ cells are essential for ovarian development [12, 13, 16–19]. In addition, the number of germ cells was demonstrated to contribute to sex differentiation in medaka and zebrafish [20–22]. Therefore, the idea suggests that germ cells might play an active role in regulating sex determination and gonad differentiation. However, the other one, originating from the observations in red-eared turtle, loach and goldfish, argued that germ cells were not primary for sexual dimorphic gonadal structures, and the number of germ cells did not alter sexual dimorphic gonad development in goldfish [23–25]. Thus, germ cells might play a passive role in sex determination and gonad differentiation in these vertebrates. Thereby, there might be two distinct functional models of germ cells on sex determination and gonad differentiation in sexual reproduction vertebrates. However, the roles of germ cells are unknown in unisexual animals, and it is also unclear whether the functional models in sexual reproduction animals are common in unisexual animals.

In this study, we have utilized consistent genetic background of gynogenetic *Carassius gibelio* created by the unisexual reproduction, and established a germ cell-depleted gonad model in the original all-ovary development by morpholino-mediated knockdown of *dead end* (Fig. 1). Subsequently, we have examined and observed significant gonadal and histological structure changes during gonad differentiation in the germ cell-depleted gynogenetic individuals (Fig. 2). Moreover, we have performed comprehensive and comparative transcriptome analysis, and revealed a complete alteration of sex-biased gene expression in which thereby leads to up-regulation of testis-biased genes and down-regulation of ovary-biased genes in the germ cell-depleted gonads of gynogenetic *Carassius gibelio* (Fig. 3 and Table 1). Through comparing dynamic expression patterns of several kinds of sex-related marker genes by transcriptomic analysis and RT-PCR detection, we have demonstrated that some testicular differentiation-related genes, such as *dmrt1*, *sox9a* and *amh*, and steroidgenic androgen-related genes, such as *hsd3b*, *hsd11b2* and *cyp17a*, are significantly up-regulated, whereas ovary differentiation-related genes including *cyp19a1a* and *foxl2*, and some ovary marker genes including *gdf9*, *h2af1o* and *zp3* are severely suppressed in the germ cell-depleted gonads (Fig. 4). Based on these finding, we have confirmed that unisexual gynogenetic embryos remain keeping male sex determination information in the genome [41]. As gonadal differentiation and development, once the leading roles of germ cells are removed from the gonads, the male sex determination genes and testis differentiation-related genes are reactivated to develop testis-like structures, just like in the germ cell-depleted gonads.

Similar to the first functional model of germ cells in sexual reproduction vertebrates, we have observed the occurrence of phenotypic masculinization and female-to-male sex reversal in the germ cell-depleted gynogenetic adults (Fig. 5). Especially in the 3-year-old germ cell-depleted gynogenetic adults, some tube-like testicular gonads have become thick and partially enlarged along with their growth and development, in which some spermatogenic cyst-like structures and the supporting somatic cells, such as Sertoli cells and Leydig cells are further differentiated. Significantly, some Sertoli cell marker genes and steroidgenic androgen enzyme genes have been confirmed to express in the 3-year-old germ cell-depleted testis-like gonads (Fig. 6). The above data have clearly demonstrated that germ cells play a leading role on gonad differentiation and sexual dimorphism in the gynogenetic *Carassius gibelio* with consistent genetic background, and the germ cell-depleted adults are sterile all-males and develop testicular gonadal structures with testicular somatic gene expression.

**Fig. 7:**
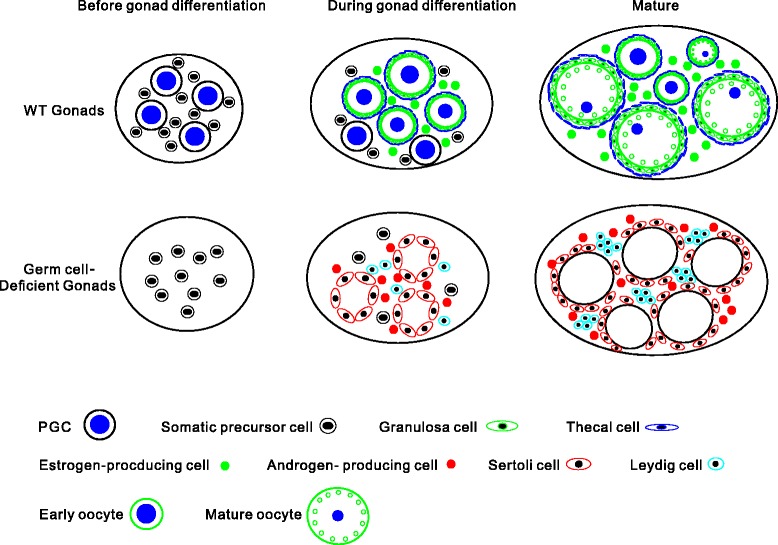
A schematic diagram of the hypothesized model for the leading role of germ cells on gonadal differentiation in the gynogenetic fish. The process of gonadal development is divided into three stages: before gonadal differentiation, during gonadal differentiation and mature. Before gonadal differentiation, there are PGCs and somatic precursor cells in WT gonads, whereas no PGCs in germ cell-deficient gonads. During gonadal differentiation, there are PGCs and somatic precursor cells, and early oocytes with granulosa cells and thecal cells on their surface, and estrogen-producing cells in WT gonads; however, in the germ cell-deficient gonads, there are somatic precursor cells, Sertoli cells, Leydig cells and androgen-producing cells. At mature stage, there are many mature occytes and several early occytes, granulosa cells and thecal cells surround around the occytes, and estrogen-producing cells are distributed among oocytes in WT gonads; whereas, there are many Sertoli cells arrayed into circles, Leydig cells and androgen-producing cells in the germ cell-deficient gonads

Based on the current findings, a hypothesized model for the leading role of germ cells on gonadal differentiation is proposed in the gynogenetic *Carassius gibelio*. As shown in Fig. 7, in WT gynogenetic gonads, an interaction between primordial germ cells and somatic precursor cells occurs in primordial gonads. As primordial germ cells develop into different stage oocytes, the primordial germ cells and oocytes enhance ovary differentiation-related gene expression, and differentiate the somatic precursor cells into estrogen-producing cells, granulosa cells and thecal cells. It is the interaction of germ cells as a leading role that promotes oogenesis, ovarian differentiation and oocyte maturation. When primordial germ cells are deleted from the primordial gonads, however, the somatic precursors in the germ cell-deficent gonads lose the leading role of germ cells, and some testis differentiation-related genes are activated to express and thereby to differentiate into testicular tissue cells, such as androgen-producing cells, Sertoli cells and Leydig cells, and finally develop into testis-like gonads in which empty spermatogenic cysts without any germ cells are surrounded by Sertoli cells and Leydig cells.

## Conclusions

Finally, our results have confirmed that unisexual gynogenetic fish remains keeping male sex determination information in the genome, and germ cell depletion completely alters sex-biased gene expression, and results in occurrence of sterile all-males with testis-like gonads and secondary sex characteristics in gynogenetic *Carassius gibelio*.

## Methods

### Source of fish

*Carassius gibelio* were cultured at Guanqiao experimental station, Institute of Hydrobiology, Chinese academy of sciences. Inducing spawning and embryos culture was performed as described previously [61]. All experiments in this research were performed according to the permit guidelines established by the Institute of Hydrobiology, Chinese Academy of Sciences, and the experimental protocols were approved by the animal care and use committee of Institute of Hydrobiology, Chinese Academy of Sciences.

### Knockdown *dnd* to generate germ-cell deficient *Carassius gibelio*

*Dnd*-MO: (*dnd*-MO: 5’- AGCTGCTGTCCCTCCATACCGCTGT-3’), and control morpholino (Con-MO, 5’ AGgTcCTGTgCCTCCATAgCcCTGT-3’) were designed and synthesized from Gene Tools as described previously [62]. To determine the optimum dosage of the *dnd*-MO, gynogenetic one-cell stage embryos were injected with various dosages (250 pg, 500 pg, 1000 pg or 2000 pg) of *dnd*-MO or 2000 pg Con-MO as control, and embryos were collected at 24 h post fertilization, and the efficiency of *dnd*-MO was examined by whole mount *in situ* hybridization analysis of *vasa*-positive cells numbers in each embryo.

### Whole mount *in situ* hybridization

The migration of PGCs during embryogenesis was examined in wild type and *dnd*-MO injected embryos by detection of *vasa* mRNA using whole mount *in situ* hybridization (WISH). Embryos from 4 cells stage to 36 hpf were collected and fixed in 4 % PFA in PBS at 4 °C overnight and stored in 100 % methanol at -20 °C. *Vasa* mRNA was detected by a digoxigenin labelled antisense *vasa* probe with 1454 bp fragment containing 3’-UTR region. *Dnd* mRNA was detected using a digoxigenin labelled antisense *dnd* probe with 700 bp fragment containing its 3’-UTR. Whole mount *in situ* hybridization was performed as previously described [63, 64].

### Histology and Immunofluorescence

Gonads were carefully anatomized in *Carassius gibelio* at various developmental stages, and fixed in 4 % PFA over night at 4 °C. Samples were dehydrated and embedded in paraffin, and was cut into 4 μm sections. Hematoxylin-Eosin staining is performed as previously described [43]. Immunofluorescence staining germ cells by *Cag*Vasa antibody was performed as previously described [42, 61].

### Relative Real-Time PCR

RT-PCR was performed as described previously [65]. Briefly, 94 °C (2 min) for heat denaturing, followed by 40 cycles of 94 °C (15 s), 57 °C (15 s), 72 °C (20 s), and additional 72 °C (2 min). *β-actin* and *Rpl13a* were as internal control (primer sequences in Additional file 9: Table S5). All the samples were analyzed in triplicates, and relative expression level of target gene was calculated with the 2^—ΔΔCT^ methods (Fig. 4) and 2^—ΔCT^ methods (Fig. 6i).

### RNA isolation and illumine RNA-sequencing

Total RNA was extracted from different developmental stage gonads (100 gonads/each *dnd*-MO or control sample) by using RNeasy Mini Kit (Qiagen 74104) according to the manufacture’s protocols. RNA quality and quantity were determined by measuring the 260/280 nm absorbance ratio using a Nanodrop® ND-2000 spectrophotometer (LabTech, Holliston, MA, USA) and Technologies 2100 Bioanalyzer (Agilent Tech nologies). The majority of the samples had an RNA Integrity Number (RIN) value higher ≥8 and 28S:18S ≥2. And 10 μg total RNA were enriched mRNA by oligo (dT) to establish cDNA library as described [66]. The library products were sequenced via Illumina HiSeqTM 2000. The gene expression level was calculated by using FPKM (comprehensive transcriptome of mature testis or ovary) or RPKM (comparative transcriptome of germ cell-depleted gonads and WT gonads at 25, 35, 45 and 60 dph) methods.

### Transcriptome assembly and annotation

Clean reads were mapped to reference sequences or reference gene set using SOAP aligner/SOAP2 [67]. For the assembly, we pooled clean reads of all samples and utilized three different trials by Trinity [68]. No more than 2 mismatches were allowed in the alignment. Unigene sequences were firstly aligned by blastx to protein databases like NR, Swiss-Prot and KEGG and then aligned by blastn to nucleotide databases nt (e-value < 0.00001), retrieving proteins with the highest sequence similarity with the given unigenes along with their protein functional annotations. Blastx was done in parallel using NOBlast. The output was used in Blast2GO, where gene ontology terms were retrieved and assigned to the transcripts [69]. The GO categorization of all DEGs covers three hierarchies: cellular component, molecular function, and biological process, and GO enrichment analysis of the differentially expressed genes was applied by GOseq R package. GO terms with corrected P-value less than 0.05 were considered significantly enriched by differentially expressed genes.

### Differential expression analysis

The analysis of differentially expressed gene between two samples was performed using the DEGseq R package. FDR (false discovery rate) was used to determine the threshold of P value in multiple analyses. In this study, statistical analysis of DEGs was performed using“FDR ≤ 0.001 and the absolute value of log2 fold change ≥ 1” as the threshold to judge the significance of gene expression difference [70, 71].

### Availability of supporting data

The associated sequence data has been deposited in National Center for Biotechnology Information (NCBI). The sequence information was descripted in Additional file 10: Table S6.

## Additional files

Additional file 1: Figure S1. A comparison among DND Protein of different vertebrates. Multiple sequences alignment of DND orthologs of *Carassius gibleio*, *Carassius auratus*, *Danio rerio*, *Misgurnus anguillicaudatus*, *Salmo salar*, *Oncorhynchus mykiss*, *Oryzias latipes*, *Oreochromis niloticus*, *Homo sapiens*, *Xenopus laevis*, *Gallus gallus* and *Mus musculus*. The RNA recognition motif (RRM) is framed in red and the identities relative to *Cag*DND are shown at the end of each sequence. (PDF 1175 kb) Additional file 2: Figure S2.
*Dnd* is a conserved maternal germ cell marker. (A-E) Expression pattern of *dnd* mRNA during embryogenesis. (A) 4 cells stage embryo, (B) 32 cells stage embryo, (C) 256 cells stage embryo, (D) 4 somite stage embryo, (E) 24 hpf embryo. (F-H) Detection of *vasa*–positive cells in 36 hpf embryos. (F) The embryo injected with con-MO, (G) The embryo injected with *dnd*-MO, and (H) The embryo injected *dnd*-MO+ *dnd* mRNA. (I) Average number of *vasa*-positive cells in 24 hpf embryos injected with various dosages of *dnd-*MO. (PDF 217 kb) Additional file 3: Table S1. The observed data from WT, con-MO and *dnd*-MO embryos at 24 hpf. (PDF 46 kb) Additional file 4: Figure S3. A digital analysis of differentially expression genes (DEGs) between the germ cell-depleted gonads and WT gonads at 25 dph (A), 35 dph (B), 45 dph (C), 60 dph (D) and between normal mature testis and ovary (E). The scattered plot indicates the compared results of log transformed gene expression levels and differentially expressed genes. Up-regulated genes are shown red, down-regulated genes are shown green, while not differentially expressed genes (Not DEGs) are in blue. (F) Venn diagram shows testis-biased genes and ovary-biased genes in mature testis and ovary. (PDF 312 kb) Additional file 5: Table S2. DEGs between germ cell-depleted gynogenetic gonads and WT gynogenetic gonads at 25, 35, 45 and 60 dph, respectively. (XLSX 5697 kb) Additional file 6: Figure S4. Histogram presentation summarizing gene ontology classification of the DEGs in germ cell-depleted gonads in comparison with WT gonads at 25 dph (A), 35 dph (B), 45 dph (C) and 60 dph (D). (PDF 883 kb) Additional file 7: Table S3. DEGs between mature testis and ovary. (XLSX 3580 kb) Additional file 8: Table S4. The secondary sex characteristics of 1-year-old WT and *dnd*-MO adults. (PDF 44 kb) Additional file 9: Table S5. Primers for RT-PCR (PDF 70 kb) Additional file 10: Table S6. Sequence information. (PDF 85 kb)

## Abbreviations

AMH: Anti-Mullerian hormone
CYP17A: Cytochrome P450, family 17, subfamily A
CYP19A1A: Cytochrome P450, family 19, subfamily A, polypeptide 1a
DEGs: Differentially expressed genes
DAZL: Deleted in azoospermia-like
DMRT1: dsx and mab-3 related transcription factor 1
DND: Dead end
FOXL2: Forkhead box L2
GDF9: Growth differentiation factor 9
H2AF1O: H2A his tone family, member 1, oocyte specific
HSD3B: Hydroxy-delta-5-steroid dehydrogenase, 3 beta
HSD11B2: Hydroxysteroid (11-beta) dehydrogenase 2
HSD17B1: Hydroxysteroid (17-beta) dehydrogenase 1
PGCs: Primordial germ cells
SF1: Nuclear receptor subfamily 5, group A, member 1a
SOX19B: SRY (sex determining region Y)-box 19b
SOX9A: SRY (sex determining region Y)-box 9a
SRD5A1: Steroid-5-alpha-reductase, alpha polypeptide 1
WT: Wild type
WT1A: Wilms tumor 1a
ZP3: Zona pellucida glycoprotein 3
