# Supplementary figures and images for: Complete depletion of primordial germ cells in an All-female fish leads to Sex-biased gene expression alteration and sterile All-male occurrence
Source: BMC Genomics. 2015 Nov 18;16:971. doi: 10.1186/s12864-015-2130-z (PMC4652418; doi:10.1186/s12864-015-2130-z)

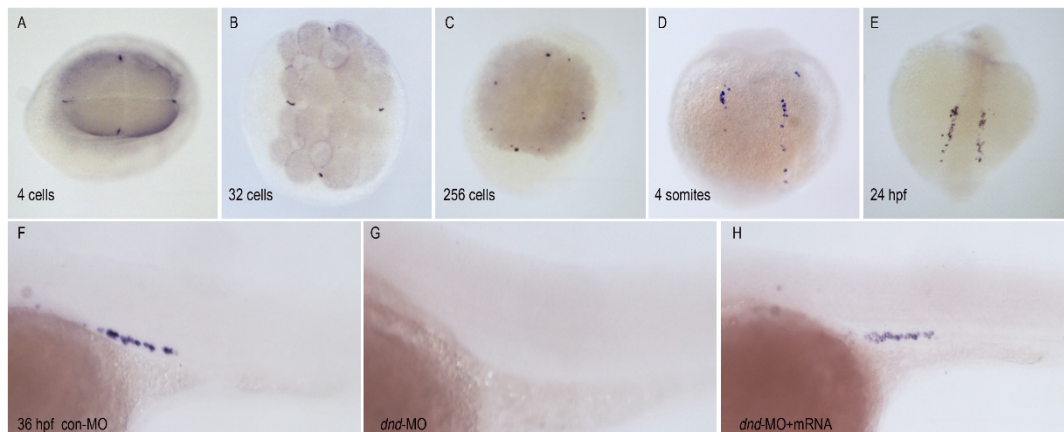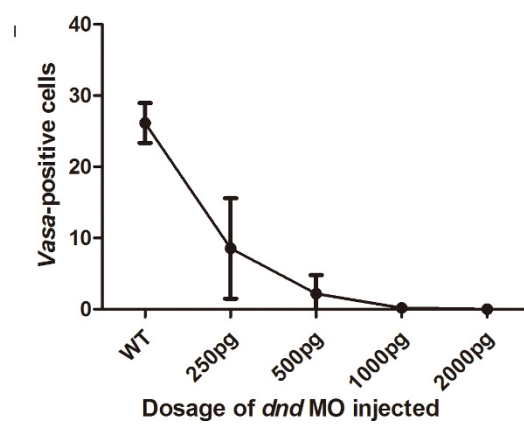

Supplement: Additional file 2: Figure S2. — Dnd is a conserved maternal germ cell marker. (A-E) Expression pattern of dnd mRNA during embryogenesis. (A) 4 cells stage embryo, (B) 32 cells stage embryo, (C) 256 cells stage embryo, (D) 4 somite stage embryo, (E) 24 hpf embryo. (F-H) Detection of vasa–positive cells in 36 hpf embryos. (F) The embryo injected with con-MO, (G) The embryo injected with dnd-MO, and (H) The embryo injected dnd-MO+ dnd mRNA. (I) Average number of vasa-positive cells in 24 hpf embryos injected with various dosages of dnd-MO. (PDF 217 kb) [file 12864_2015_2130_MOESM2_ESM.pdf]

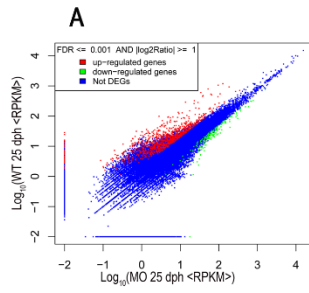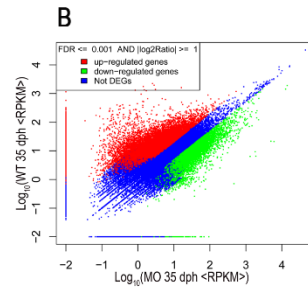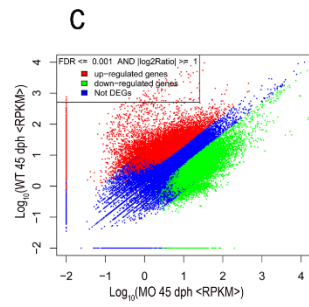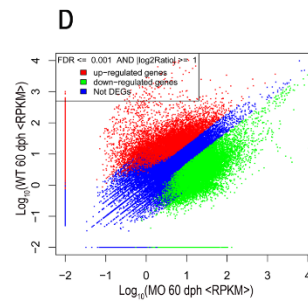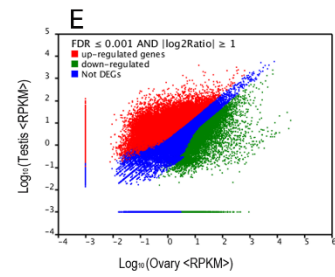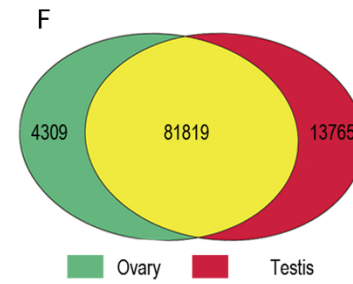

Supplement: Additional file 4: Figure S3. — A digital analysis of differentially expression genes (DEGs) between the germ cell-depleted gonads and WT gonads at 25 dph (A), 35 dph (B), 45 dph (C), 60 dph (D) and between normal mature testis and ovary (E). The scattered plot indicates the compared results of log transformed gene expression levels and differentially expressed genes. Up-regulated genes are shown red, down-regulated genes are shown green, while not differentially expressed genes (Not DEGs) are in blue. (F) Venn diagram shows testis-biased genes and ovary-biased genes in mature testis and ovary. (PDF 312 kb) [file 12864_2015_2130_MOESM4_ESM.pdf]
