# Supplementary material for: Complete depletion of primordial germ cells in an All-female fish leads to Sex-biased gene expression alteration and sterile All-male occurrence
Source: BMC Genomics. 2015 Nov 18;16:971. doi: 10.1186/s12864-015-2130-z (PMC4652418; doi:10.1186/s12864-015-2130-z)
Supplement: Additional file 3: Table S1. — The observed data from WT, con-MO and dnd-MO embryos at 24 hpf. (PDF 46 kb) [file 12864_2015_2130_MOESM3_ESM.pdf]

	Total No. of embryos	Survival embryos	Embryos without PGCs	Embryos with normal PGCs
WT	150	138	0	138
con-MO	150	130	0	130
dnd-MO	150	133	132	1
