# Supplementary material for: Complete depletion of primordial germ cells in an All-female fish leads to Sex-biased gene expression alteration and sterile All-male occurrence
Source: BMC Genomics. 2015 Nov 18;16:971. doi: 10.1186/s12864-015-2130-z (PMC4652418; doi:10.1186/s12864-015-2130-z)

A DEGs in *dnd*-MO gonads VS WT gonads at 25 dph

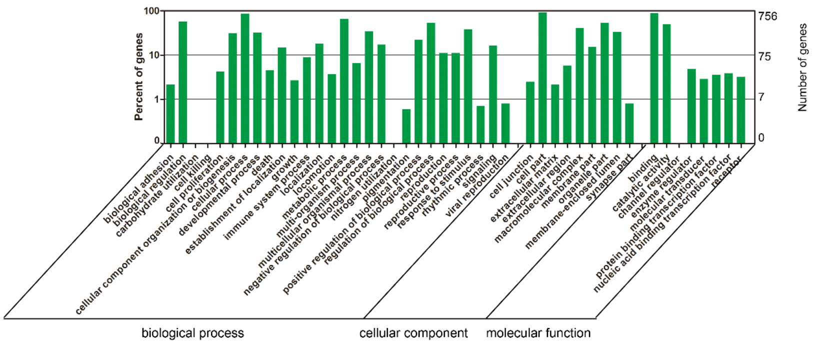

B DEGs in *dnd*-MO gonads VS WT gonads at 35 dph

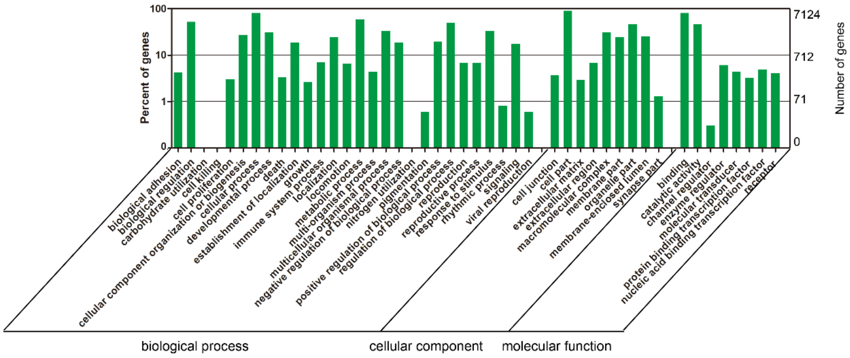

C DEGs in *dnd*-MO gonads VS WT gonads at 45 dph

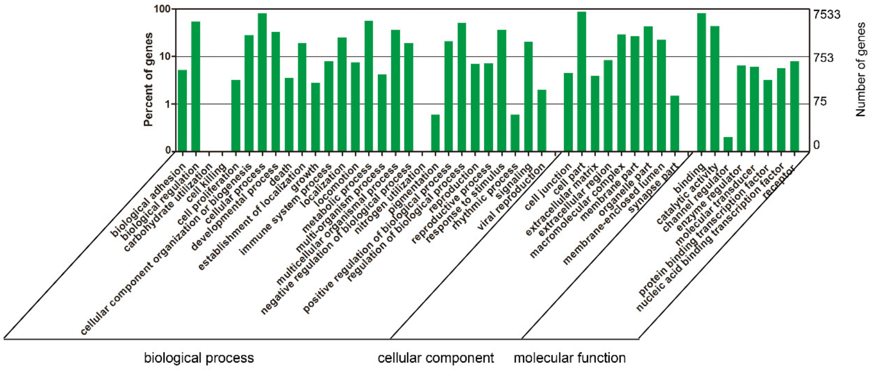

D DEGs in *dnd*-MO gonads VS WT gonads at 60 dph

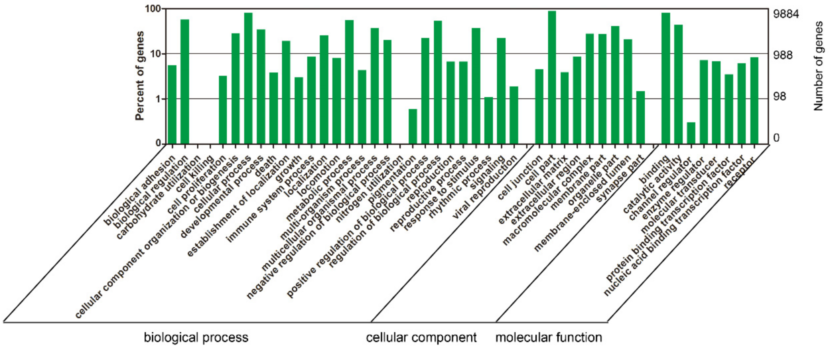

Supplement: Additional file 6: Figure S4. — Histogram presentation summarizing gene ontology classification of the DEGs in germ cell-depleted gonads in comparison with WT gonads at 25 dph (A), 35 dph (B), 45 dph (C) and 60 dph (D). (PDF 883 kb) [file 12864_2015_2130_MOESM6_ESM.pdf]
