# Supplementary material for: Complete depletion of primordial germ cells in an All-female fish leads to Sex-biased gene expression alteration and sterile All-male occurrence
Source: BMC Genomics. 2015 Nov 18;16:971. doi: 10.1186/s12864-015-2130-z (PMC4652418; doi:10.1186/s12864-015-2130-z)
Supplement: Additional file 8: Table S4. — The secondary sex characteristics of 1-year-old WT and dnd-MO adults. (PDF 44 kb) [file 12864_2015_2130_MOESM8_ESM.pdf]

	Total number	Body shape		anus		Pearl organs
		slender	fat	prolate	round	
WT	90	0	90	0	90	0
dnd-MO	85	84	1*	84	1*	84
