# Supplementary material for: Complete depletion of primordial germ cells in an All-female fish leads to Sex-biased gene expression alteration and sterile All-male occurrence
Source: BMC Genomics. 2015 Nov 18;16:971. doi: 10.1186/s12864-015-2130-z (PMC4652418; doi:10.1186/s12864-015-2130-z)
Supplement: Additional file 9: Table S5. — Primers for RT-PCR (PDF 70 kb) [file 12864_2015_2130_MOESM9_ESM.pdf]

Gene	Direction	Primer sequences (5'-3')
Vasa	Forward	GGGAAGGCAAACAATGAAGA
	Reverse	CTTGGCCTTCATCTTTCCAA
Dmrt1	Forward	GAAATGGTCGCTATAACGTG
	Reverse	GATGATGGAGTCGACAGAGA
Foxl2	Forward	ACATTAAGCTGCACAGAAACTA
	Reverse	GAGGTAGTGTCCATGAGTATC
Amh	Forward	GCCGGTTTTAGTTTTCAAC
	Reverse	AAAGAGACGATTTTTGCAC
Cyp19a1a	Forward	AACTCTCCAGAATGTGTAACCTG
	Reverse	TCCAGACACTCTCTCTATCAGTC
Cyp17a	Forward	ACCATCACCCTCCTCGTTCA
	Reverse	CACAATAAGAGAAGTTCTGA
Hsd3b	Forward	AAAAGCTGGTCAGGCTGTTG
	Reverse	AGACAGGAGGAGTAAGGGGT
Hsd11b2	Forward	TGTTCATCACGCCTTACCCT
	Reverse	CATGGCTCCAACTCATGACG
Hsd17b1	Forward	CTGCTGACTTTGGCTCTGTG
	Reverse	AGAGCAGTAGTCACCCAACC
Sox9a	Forward	GAGGAAATCAGTGAAGAACG
	Reverse	CTGAGACTGACCTGAGTGGT
β-actin	Forward	CGAGCTGTCTTCCCATCCA
	Reverse	CCAACGTAGCTGTCTTTCTG
Dnd	Forward	GAACTTCAGTGGGCAGAACC
	Reverse	GTCAGAGATCATTCGCAGCA
H2af1o	Forward	TATGTCCGGTCGCGGTAAGAA
	Reverse	AGTCCAGTGCACGAGCGGAGT
Zp3	Forward	TGACGCTCTCCACAAATCCT
	Reverse	ACCATTACAGGACCGAGCAA
Gdf9	Forward	GGTCTCAGGAGCAGCTGCTG
	Reverse	GCTTGGATGAGAGGATGGA
Wt1a	Forward	AGCTCACAACAGGCCCTAAT
	Reverse	TGGTCAGACAGCATGGATGT
Sox19b	Forward	AAATGTTGCCGTTTCAGCCT
	Reverse	ATCACTGGTCATGGCAGTCA
Rpl13a	Forward	TCTGGAGGACTGTAAGAGGTATGC
	Reverse	AGACGCACAATCTTGAGAGCAG
