# Supplementary material for: Complete depletion of primordial germ cells in an All-female fish leads to Sex-biased gene expression alteration and sterile All-male occurrence
Source: BMC Genomics. 2015 Nov 18;16:971. doi: 10.1186/s12864-015-2130-z (PMC4652418; doi:10.1186/s12864-015-2130-z)
Supplement: Additional file 10: Table S6. — Sequence information. (PDF 85 kb) [file 12864_2015_2130_MOESM10_ESM.pdf]

Sequence Information.

The transcriptome raw data of mature testis and ovary were submitted to NCBI database with accession NO. SRP056613, and the clean data of the comparative analysis in the WT and germ cell-depleted gonads were also submitted to NCBI database with accession NO. SRP056766.

A 1499-bp *dnd* cDNA was isolated; this had an ORF of 1125bp, encoding a putative 375-aa protein (KP641680). It was similar to Dnd orthologs of Goldfish (92.8%, AEX33122.1), Zebrafish (67.1%, NP_997960.1), Loach (62.4%, BAJ19134.1) and Atalantic salmon (47.4%, NP_001266060.1).

A 2103-bp *cyp17a* cDNA was isolated; this had an ORF of 1551bp, encoding a putative 517-aa protein (KP255964). It was similar to Cyp17a orthologs of Zebrafish (91%, AAI62669.1), Medaka (74%, NP_001098564.1), Rainbow trout (75%, NP_001118219.1) and Nile tilapia (72%, NP_001266694.1).

A 1468-bp *cyp19a1a* cDNA was isolated; this had an ORF of 1110 bp, encoding a putative 370-aa protein (KP255965). It was similar to Cyp19a1a orthologs of Zebrafish (91%, AAG12243.1), Medaka (71%, NP_001265808.1), Rainbow trout (76%, 228574) and Yellow catfish (77%, AAW65999.1).

A 2158-bp *hsd11b2* cDNA was isolated; this had an ORF of 1239 bp, encoding a putative 413-aa protein (KP255966). It was similar to Hsd11b2 orthologs of Zebrafish (89%, NP_997885.2), Medaka (68%,

XP_004066855.1), Rainbow trout (75%, NP_001117690.1) and Nile tilapia (67%, NP_001266686.1).

A 1050-bp *hsd17b1* cDNA was isolated; this had an ORF of 876 bp, encoding a putative 292-aa protein (KP255967). It was similar to Hsd11b2 orthologs of Zebrafish (89%, NP_997885.2), Medaka (68%, XP_004066855.1), Rainbow trout (75%, NP_001117690.1) and Nile tilapia (67%, NP_001266686.1).

A 1611-bp *foxl2* cDNA was isolated; this had an ORF of 870 bp, encoding a putative 290-aa protein (KP255968). It was similar to Foxl2 orthologs of Zebrafish (83%, XM_009298955.1), Chicken (83%, NM_001012612.1), Frog (80%, NM_001134784.1) and Mouse (87%, BC137812.1).

A 2230-bp *amh* cDNA was isolated; this had an ORF of 1689 bp, encoding a putative 563-aa protein (KP255971). It was similar to Amh orthologs of Goldfish (99%, JN578699.1), Zebrafish (79%, AY677080.2) and Medaka (71%, AB166790.1).

A 600-bp *nanos3* cDNA was isolated; this had an ORF of 498 bp, encoding a putative 166-aa protein (KP255972). It was similar to Nanos3 orthologs of Common carp (89%, AB576134.1), Zebrafish (80%, NM_131878.1), Medaka (78%, NM_001122828.1) and Rainbow trout (72%, NM_001281422.1).

A 3804-bp *tdrd7* cDNA was isolated; this had an ORF of 3216 bp, encoding a putative 1072-aa protein (KP255973). It was similar to Tdrd7 orthologs

of Zebrafish (85%, NM_001099343.1), Nile tilapia (74%, XM_005462506.1) and Medaka (72%, XM_004079730.1).

A 2271-bp *sox9a* cDNA was isolated; this had an ORF of 1269 bp, encoding a putative 423-aa protein (KP255974). It was similar to Sox9a orthologs of Zebrafish (71%, NP_571718.1), Nile tilapia (71%, XP_003450167.1), Medaka (72%, AAX62151.1) and Rainbow trout (70%, NP_001117651.1).

A 986-bp *hsd3b* partial cDNA was isolated, encoding 328-aa (KP641679). It was similar to Hsd3b orthologs of Zebrafish (86%, XP_694204.3), Rainbow trout (74%, AAB31733.1), Nile tilapia (68%, NP_001266438.1) and Medaka (68%, NP_001131037.1).
